# Role of PCSK9 inhibition during the inflammatory stage of SARS-COV-2: an updated review

**DOI:** 10.1097/MS9.0000000000001601

**Published:** 2024-01-03

**Authors:** Hina Arsh, FNU Manoj Kumar, FNU Simran, Sweta Tamang, Mahboob ur Rehman, Gulfam Ahmed, Masood Khan, Jahanzeb Malik, Amin Mehmoodi

**Affiliations:** a Department of Medicine, THQ Hospital, Pasrur; b Department of Medicine, Jinnah Sindh Medical College, Karachi; c Department of Cardiology, Pakistan Institute of Medical Sciences; d Department of Cardiovascular Medicine, Cardiovascular Analytics Group, Islamabad; e Department of Medicine, Muhammad Hospital, Lahore; f Department of Cardiology, Armed Forces Institute of Cardiology, Rawalpindi, Pakistan; g Department of Medicine, Nepal Medical College and Teaching Hospital, Kathmandu, Nepal; h Department of Medicine, Ibn e Seena Hospital, Kabul, Afghanistan

**Keywords:** cardiovascular protection, COVID-19, inflammation, PCSK9 inhibition, thrombosis

## Abstract

The potential role of proprotein convertase subtilisin/kexin type 9 (PCSK9) inhibition in the management of COVID-19 and other medical conditions has emerged as an intriguing area of research. PCSK9 is primarily known for its impact on cholesterol metabolism, but recent studies have unveiled its involvement in various physiological processes, including inflammation, immune regulation, and thrombosis. In this abstract, the authors review the rationale and potential implications of PCSK9 inhibition during the inflammatory stage of SARS-CoV-2 infection. Severe cases of COVID-19 are characterized by an uncontrolled inflammatory response, often referred to as the cytokine storm, which can lead to widespread tissue damage and organ failure. Preclinical studies suggest that PCSK9 inhibition could dampen this inflammatory cascade by reducing the production of pro-inflammatory cytokines. Additionally, PCSK9 inhibition may protect against acute respiratory distress syndrome (ARDS) through its effects on lung injury and inflammation. COVID-19 has been linked to an increased risk of cardiovascular complications, especially in patients with pre-existing cardiovascular conditions or dyslipidemia. PCSK9 inhibitors are known for their ability to lower low-density lipoprotein (LDL) cholesterol levels by enhancing the recycling of LDL receptors in the liver. By reducing LDL cholesterol, PCSK9 inhibition might protect blood vessels from further damage and lower the risk of atherosclerotic plaque formation. Moreover, PCSK9 inhibitors have shown potential antithrombotic effects in preclinical studies, making them a potential avenue to mitigate the increased risk of coagulation disorders and thrombotic events observed in COVID-19. While the potential implications of PCSK9 inhibition are promising, safety considerations and possible risks need careful evaluation. Hypocholesterolemia, drug interactions, and long-term safety are some of the key concerns that should be addressed. Clinical trials are needed to establish the efficacy and safety of PCSK9 inhibitors in COVID-19 patients and to determine the optimal timing and dosing for treatment. Future research opportunities encompass investigating the immune response, evaluating long-term safety, exploring combination therapy possibilities, and advancing personalized medicine approaches. Collaborative efforts from researchers, clinicians, and policymakers are essential to fully harness the therapeutic potential of PCSK9 inhibition and translate these findings into meaningful clinical outcomes.

## Introduction

Highlights

- The potential role of proprotein convertase subtilisin/kexin type 9 (PCSK9) inhibition in the management of COVID-19 and other medical conditions has emerged as an intriguing area of research.
- PCSK9 is primarily known for its impact on cholesterol metabolism, but recent studies have unveiled its involvement in various physiological processes, including inflammation, immune regulation, and thrombosis.
- Preclinical studies suggest that PCSK9 inhibition could dampen this inflammatory cascade by reducing the production of pro-inflammatory cytokines.
- PCSK9 inhibitors have shown potential antithrombotic effects in preclinical studies, making them a potential avenue to mitigate the increased risk of coagulation disorders and thrombotic events observed in COVID-19.
- Clinical trials are needed to establish the efficacy and safety of PCSK9 inhibitors in COVID-19 patients and to determine the optimal timing and dosing for treatment.

The COVID-19 pandemic caused by the SARS-CoV-2 has led to significant morbidity and mortality worldwide^1^. One of the most severe complications of COVID-19 is acute respiratory distress syndrome (ARDS) and systemic inflammation, which can escalate into a potentially life-threatening cytokine storm^2^. The extent of immune dysregulation is a crucial determinant of patient outcomes, with greater dysregulation associated with worse prognoses and increased mortality. In this context, targeting key components of the inflammatory response during COVID-19 holds promising potential for effective therapeutic interventions, especially for critically ill patients^3^. Among the various inflammatory drivers in COVID-19, interleukin (IL)-6 has emerged as a major contributor, with elevated levels of IL-6 being predictive of a more severe disease course^4^. Proprotein convertase subtilisin/kexin type 9 (PCSK9) is an enzyme known for its role in regulating low-density lipoprotein (LDL) receptors, and it has been associated with vascular inflammation^5^. During acute inflammation, there is an accumulation of oxidized LDL and apolipoprotein B, which leads to the generation of cholesterol crystals in macrophages. These cholesterol crystals activate the inflammasome complex, triggering the release of inflammatory cytokines. Interestingly, PCSK9 has been shown to directly activate pro-inflammatory signalling pathways, leading to increased cytokine production^6^. Recent studies have also highlighted the impact of PCSK9 on the immune response in patients with septic shock, wherein lower PCSK9 function was associated with reduced inflammatory responses and improved overall outcomes^7^. Additionally, individuals with a loss-of-function genotype for PCSK9 have shown lower PCSK9 levels and enhanced resolution of infections. Given the potential influence of PCSK9 on inflammatory pathways and its role in lipid metabolism, researchers have explored the use of PCSK9 inhibitors as potential therapeutic agents in COVID-19^8^. PCSK9 inhibitors are potent lipid-lowering drugs known to reduce LDL cholesterol (LDL-C) levels, and they may also have direct effects on PCSK9-mediated inflammatory pathways^9^. Both experimental and clinical evidence suggest that PCSK9 inhibitors could exert anti-inflammatory effects by interfering with the IL-6–mediated inflammatory pathway triggered by PCSK9^10^. These findings have sparked interest in investigating the role of PCSK9 inhibition as a potential treatment strategy during the inflammatory stage of SARS-CoV-2 infection. In this updated review, we aim to delve into the current understanding of the role of PCSK9 inhibition in mitigating the inflammatory response during COVID-19. By examining the experimental and clinical evidence, we seek to explore the potential benefits of PCSK9 inhibitors as a novel therapeutic approach in managing the inflammatory complications of SARS-CoV-2 infection, with a focus on critically ill patients. As the global health community continues its relentless efforts to combat the COVID-19 pandemic, a comprehensive assessment of the role of PCSK9 inhibition in modulating the immune response may provide valuable insights into the development of more effective treatment strategies for severe cases of COVID-19.

## Methods

A comprehensive literature search was conducted to identify relevant studies on the role of PCSK9 inhibition during the inflammatory stage of SARS-CoV-2 infection. Electronic databases, including PubMed, Embase, Scopus, and Web of Science, were searched. The search terms included combinations of keywords related to PCSK9, COVID-19, inflammation, cytokine storm, IL-6, and PCSK9 inhibitors. The search was restricted to studies published in English up to the date of the search, with a focus on articles published within the last 2 years. Two independent reviewers screened the search results for eligibility based on predefined inclusion and exclusion criteria. Inclusion criteria included studies investigating the impact of PCSK9 inhibition on the inflammatory response during COVID-19, both experimental and clinical studies. Studies assessing PCSK9 inhibitors in other inflammatory conditions were also considered if they provided mechanism of PCSK9 inhibition on inflammation and immune regulation. Data from the selected studies were independently extracted by two reviewers using a standardized data extraction form. The was presented in the form of narrative synthesis.

### PCSK9 function

PCSK9 plays a crucial role in regulating cholesterol metabolism, specifically through its impact on the expression and function of the LDLR^11^. Disruptions in PCSK9 activity can significantly affect plasma cholesterol levels, contributing to the development of cardiovascular diseases (CVD)^12^. PCSK9 is encoded by the PCSK9 gene found on chromosomes 1p32.3 in humans and 4C7 in mice^13^. The gene consists of thirteen exons encoding a 692-amino acid PCSK9 protein in humans and twelve exons encoding a 694-amino acid PCSK9 protein in mice^14^. Additionally, PCSK9 shows high conservation across various mammalian species, suggesting its vital role in cholesterol regulation. The PCSK9 protein comprises four main domains: the signal peptide, prodomain, catalytic domain, and C-terminal domain^15^. During its maturation, PCSK9 undergoes autocleavage by its catalytic domain, generating the mature enzyme. The prodomain is necessary for PCSK9’s maturation and secretion, as it associates with the catalytic domain and inhibits its activity. PCSK9 primarily acts as a negative regulator of LDLR by binding to it on the cell surface and facilitating its internalization^16^. Once internalized, the PCSK9-LDLR complex remains in acidic endosomes, preventing LDLR recycling and targeting it for lysosomal degradation^17^. This leads to reduced LDL-C clearance from the bloodstream and elevated plasma LDL-C levels. Apart from the liver, PCSK9 is expressed in various extra-hepatic tissues, such as vascular smooth muscle cells, macrophages, endothelial cells, pancreatic beta cells, and the central nervous system^18^. Its function in these tissues may vary, indicating cell and tissue-specific roles. PCSK9 also affects triglyceride-rich lipoproteins, such as very VLDL and chylomicrons^19^. It can influence the production and secretion of VLDL and modulate the metabolism of intestinal chylomicrons, though its impact on plasma triglyceride levels is relatively modest compared to its effect on LDL-C. PCSK9 has garnered significant interest in its role in regulating lipoprotein(a) (Lp(a)), a lipoprotein-associated with an increased risk of CVD^20^. Inhibition of PCSK9 has been found to reduce plasma Lp(a) levels, providing a potential therapeutic avenue for managing elevated Lp(a) and lowering CVD risk^21^. PCSK9’s actions on lipoprotein metabolism involve a complex interplay between its extracellular and intracellular pathways. The extracellular pathway predominantly regulates LDLR-mediated catabolism, while the intracellular pathway can modulate apoB secretion, impacting VLDL and chylomicron metabolism^22^.

### PCSK9’s role in LDLR regulation

PCSK9-LDLR binding and endocytosis are crucial steps in PCSK9’s role as a regulator of cholesterol metabolism^16^. The interaction between PCSK9 and LDLR on the cell surface is a highly specific and tightly regulated process that influences LDL-C clearance and plasma cholesterol levels. The binding of PCSK9 to LDLR occurs through specific domains on both proteins. PCSK9 possesses a catalytic domain that plays a critical role in recognizing and binding to the LDLR^23^. Meanwhile, LDLR contains an epidermal growth factor precursor homology domain A (EGF-A), which serves as the binding site for PCSK9. The process of binding begins with the recognition of LDLR by PCSK9 through the interaction of their respective domains^24^. This interaction is characterized by specific amino acid residues in the catalytic domain of PCSK9, which align with complementary residues in the EGF-A domain of LDLR. Following the binding of PCSK9 to LDLR, the complex is internalized into the cell through endocytosis. Endocytosis is a cellular process that involves the invagination of the cell membrane, leading to the formation of vesicles containing extracellular materials, including the PCSK9-LDLR complex^25^. The process of endocytosis allows the cell to internalize and transport various molecules from the cell surface to the intracellular compartments. In the context of PCSK9 and LDLR, endocytosis is a critical mechanism for regulating LDLR levels on the cell surface and determining the fate of LDLR in cholesterol metabolism. Once internalized, the PCSK9-LDLR complex is transported through the endosomal network within the cell. The complex eventually reaches acidic endosomes, which are compartments characterized by their low pH environment. The acidic pH of the endosomes triggers conformational changes in the PCSK9-LDLR complex, facilitating the dissociation of PCSK9 from LDLR. This release of PCSK9 from LDLR is a crucial step that sets the stage for further LDLR regulation^26^. Upon dissociation from PCSK9, LDLR can take one of two paths within the endosomal system: recycling back to the cell surface or being directed to lysosomes for degradation. In the absence of PCSK9, LDLR typically undergoes recycling, allowing it to return to the cell surface and continue its role in capturing LDL-C from the bloodstream. However, in the presence of PCSK9, recycling is inhibited, and LDLR is targeted for lysosomal degradation^27^. The PCSK9-LDLR complex in acidic endosomes is recognized by lysosomal targeting signals, leading to the recruitment of lysosomal enzymes^28^. These enzymes promote the ubiquitination of LDLR, marking it for degradation within the lysosomes. The degradation of LDLR in lysosomes, facilitated by PCSK9, results in a reduction of LDLR available on the cell surface. This reduced LDLR expression decreases the uptake and clearance of LDL-C particles from the bloodstream, leading to elevated plasma LDL-C levels. As a consequence, dysregulated PCSK9 activity can contribute to hypercholesterolaemia and increase the risk of developing cardiovascular diseases, such as atherosclerosis and coronary artery disease^29^.

### Rationale for PCSK9 inhibition during SARS-CoV-2 infection

The rationale for PCSK9 inhibition during SARS-CoV-2 infection stems from the interplay between lipid metabolism, inflammation, and cardiovascular complications observed in COVID-19 patients. PCSK9 (Proprotein Convertase Subtilisin/Kexin Type 9) is a protein primarily known for its role in regulating cholesterol levels by promoting the degradation of LDLRs in the liver. However, recent research has uncovered additional functions of PCSK9 that go beyond lipid metabolism, indicating its potential involvement in inflammatory processes and immune responses.

#### Dyslipidemia and COVID-19

Dyslipidemia is a common metabolic disorder characterized by abnormal levels of lipids in the bloodstream. It can involve elevated levels of LDL-C, and triglycerides, as well as reduced levels of HDL-C^30^. These lipid imbalances can lead to the development of atherosclerosis, a condition where plaque buildup occurs on the inner walls of arteries. COVID-19 can directly impact the cardiovascular system, leading to a range of cardiovascular complications. The virus can infect endothelial cells that line the blood vessels, causing endothelial dysfunction, inflammation, and damage^31^. This process can trigger a cascade of events, including platelet activation, coagulation abnormalities, and the formation of blood clots. The combination of viral effects and systemic inflammation can exacerbate existing cardiovascular conditions, such as dyslipidemia and atherosclerosis, leading to increased cardiovascular risk in COVID-19 patients^32^. Dyslipidemia has been associated with an increased risk of severe COVID-19 outcomes. Elevated LDL cholesterol levels are considered a major risk factor for atherosclerotic plaque formation. The presence of underlying atherosclerosis can lead to unstable plaques that are more prone to rupture, resulting in acute cardiovascular events, such as heart attacks or strokes, in COVID-19 patients^33^. Moreover, dyslipidemia may contribute to an excessive inflammatory response to COVID-19. Dyslipidemia can activate immune cells and promote the release of pro-inflammatory cytokines, thereby exacerbating the cytokine storm observed in severe cases of COVID-19^34^. This hyperinflammatory state can lead to a vicious cycle of tissue damage and inflammation, further increasing the risk of cardiovascular complications.

Optimal management of dyslipidemia is essential to reduce the risk of cardiovascular complications in COVID-19 patients^35^. Some studies suggest that statins might have a beneficial impact on COVID-19 outcomes by dampening the inflammatory response^36^. However, more research is needed to establish a definitive link between statin use and COVID-19 outcomes. In COVID-19 patients with dyslipidemia, managing cardiovascular risk requires a comprehensive and individualized approach. However, there are challenges in ensuring optimal lipid management during the pandemic. Access to healthcare services may be limited due to overwhelming demands on healthcare systems, and some patients may be hesitant to seek medical care due to infection concerns^37^. Additionally, potential drug interactions and safety considerations should be carefully evaluated when prescribing lipid-lowering medications to COVID-19 patients receiving other treatments or therapies.

#### PCSK9 and inflammation

Several studies have indicated that PCSK9 can influence the inflammatory milieu through various mechanisms^38^. One key observation is that PCSK9 promotes the production of pro-inflammatory cytokines, including interleukin-6 (IL-6) and tumour necrosis factor-alpha (TNF-alpha)^39^. These cytokines are crucial mediators of the immune response and play significant roles in initiating and sustaining inflammation. For instance, a study found that PCSK9 expression was increased in the aortic tissues of mice exposed to a high-fat diet^40^. This upregulation of PCSK9 was associated with increased levels of pro-inflammatory cytokines, suggesting a potential link between PCSK9 and inflammation in the context of atherosclerosis, a chronic inflammatory condition. Beyond its effects on cytokine production, PCSK9 may also impact immune cell function^41^. Immune cells play a central role in orchestrating the immune response during infections and inflammatory conditions. Studies have shown that PCSK9 can influence the differentiation and activation of immune cells, such as T cells and macrophages^42^. This suggests that PCSK9 may promote the recruitment and activation of pro-inflammatory immune cells, contributing to the inflammatory environment in atherosclerosis. Considering the role of PCSK9 in inflammation and immune responses, it is reasonable to explore its potential relevance in the context of viral infections, including COVID-19. In COVID-19, the immune response is a critical determinant of disease severity^43^. The virus can trigger a dysregulated immune response characterized by an excessive release of pro-inflammatory cytokines, known as the cytokine storm^44^. This immune dysregulation contributes to tissue damage, organ dysfunction, and severe clinical outcomes in some COVID-19 patients. As PCSK9 has been linked to the production of pro-inflammatory cytokines, it is possible that PCSK9 may contribute to the cytokine storm observed in severe COVID-19 cases. Targeting PCSK9 could be a potential strategy to modulate the inflammatory response and potentially alleviate the severity of COVID-19^45^. The growing understanding of PCSK9’s role in inflammation and immune responses opens up potential therapeutic implications. In addition to its established role in cholesterol metabolism, PCSK9 inhibitors might have additional benefits in certain inflammatory conditions, including atherosclerosis and potentially COVID-19. However, it is essential to approach the use of PCSK9 inhibitors cautiously, considering the complexity of immune responses and potential off-target effects. Further research, including clinical trials, is necessary to explore the safety and efficacy of PCSK9 inhibitors as immunomodulatory agents in various inflammatory conditions, including COVID-19.

#### Enhanced inflammatory response in COVID-19

**Figure 1:**
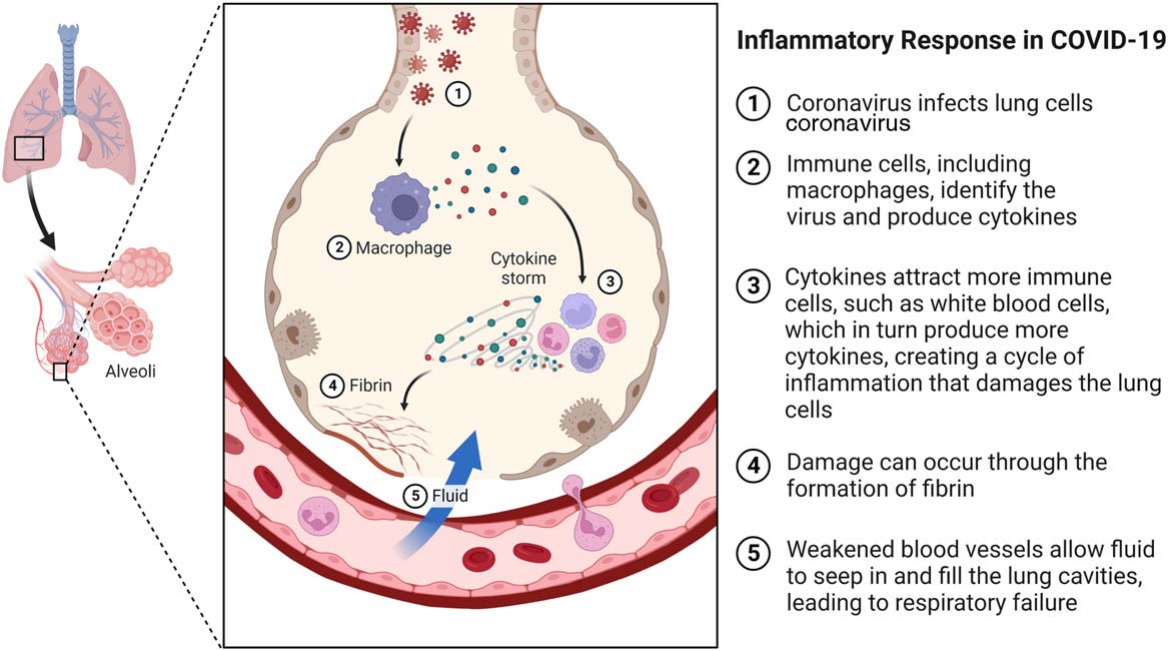
Inflammatory response of COVID-19.

The inflammatory response plays a critical role in the body’s defense against infections. However, in some severe cases of COVID-19, the immune response becomes dysregulated, leading to an exaggerated and uncontrolled release of pro-inflammatory cytokines, commonly referred to as a cytokine storm^46^. This cytokine storm is a key contributor to the development of ARDS and multi-organ failure, two of the most severe and life-threatening complications of COVID-19^47^. The cytokine storm is characterized by an overwhelming release of pro-inflammatory cytokines, such as interleukin-6 (IL-6), tumour necrosis factor-alpha (TNF-alpha), interleukin-1 beta (IL-1β), and others^48^. This excessive cytokine production can lead to widespread inflammation, tissue damage, and disruption of normal physiological processes. In severe cases of COVID-19, the immune system’s response to the SARS-CoV-2 virus can become dysregulated, resulting in a massive and uncontrolled cytokine release. This cytokine storm can cause severe damage to the lungs, leading to ARDS, where the lungs become inflamed and filled with fluid, severely impairing oxygen exchange^49^. Additionally, systemic inflammation can lead to damage in other organs, including the heart, kidneys, and liver, contributing to multi-organ failure (Fig. 1). Recent studies have highlighted the involvement of PCSK9 in the modulation of inflammation^50^. While PCSK9 is primarily known for its role in cholesterol metabolism, emerging evidence suggests that it also affects the immune system and inflammatory processes. Studies have shown that PCSK9 can promote the production of pro-inflammatory cytokines, including IL-6 and TNF-alpha, in immune cells. A study demonstrated that PCSK9 enhances the production of pro-inflammatory cytokines in human macrophages, indicating a potential link between PCSK9 and inflammation^51^.

#### Potential therapeutic benefits

By inhibiting PCSK9, we could not only improve lipid metabolism but also modulate the inflammatory response in COVID-19 patients. Lowering LDL cholesterol levels through PCSK9 inhibition might protect blood vessels from further damage, reducing the risk of cardiovascular complications in COVID-19 patients with dyslipidemia. Simultaneously, the suppression of PCSK9-mediated inflammation might help attenuate the cytokine storm and mitigate the severe immune response associated with COVID-19.

### Potential implications of PCSK9 in SARS-COV-2

**Figure 2:**
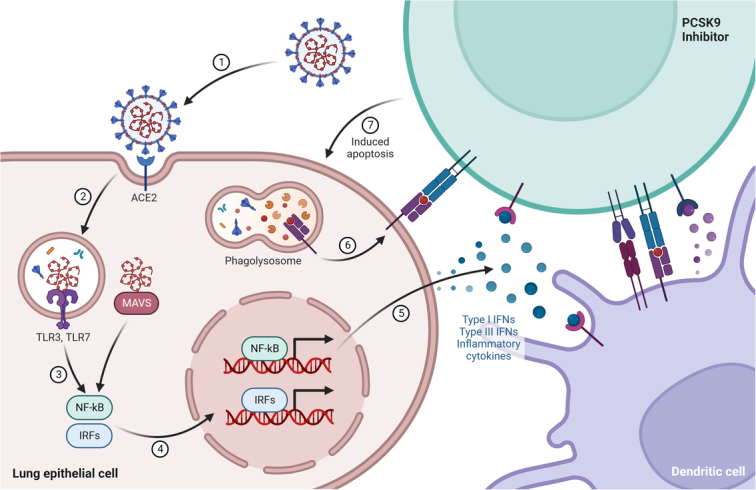
PCSK9 activates endosomal and cytoplasmic sensors, TLR3/7 and MAVS, respectively. These receptors activate interferon regulatory factors (IRFs) and NFkB to induce inflammatory cytokines, including interferons (IFN). Dendritic cells (DCs) sample antigen and migrate to lymphoid organs to prime adaptive immunity. CD8 T cells induce apoptosis after recognition of antigen on DCs or infected cells. PCSK9, proprotein convertase subtilisin/kexin type 9.

PCSK9 inhibition during the inflammatory stage of SARS-CoV-2 infection could have several potential implications, ranging from the modulation of the immune response to the protection of cardiovascular health (Fig. 2).

#### Attenuation of the cytokine storm

PCSK9 can attenuate inflammation through various mechanisms^1^. One of the key pathways is the regulation of LDLR expression. PCSK9 binds to LDLRs and targets them for degradation, reducing their availability on the cell surface. This, in turn, leads to increased LDL cholesterol levels. However, when PCSK9 is inhibited, LDLRs are protected from degradation, resulting in enhanced LDL cholesterol clearance from the blood^52^. Interestingly, LDLRs also play a role in immune cell function. Macrophages, a type of immune cell, express LDLRs and use them to internalize LDL particles. When LDLR expression is increased due to PCSK9 inhibition, macrophages take up more LDL particles^39^. This process can lead to the production of anti-inflammatory molecules and the suppression of pro-inflammatory cytokines. Additionally, PCSK9 inhibition might reduce the expression of inflammatory molecules in endothelial cells lining the blood vessels, further modulating the inflammatory response. Some animal studies have provided insight into the potential benefits of PCSK9 inhibition in the context of inflammation and acute lung injury, which are relevant to COVID-19^33^. Based on these preclinical findings, there is a hypothesis that PCSK9 inhibition might have a beneficial impact on the inflammatory stage of COVID-19^25^. By reducing pro-inflammatory cytokine production and promoting anti-inflammatory responses, PCSK9 inhibition could potentially dampen the cytokine storm, thereby mitigating the severity of the disease and improving patient outcomes^28^. While the preclinical evidence is promising, it is essential to note that the translation of findings from animal studies to human patients can be complex. Additionally, the inflammatory response to COVID-19 is highly dynamic and influenced by various factors, including viral load, host immune status, and genetic predisposition^35^. Therefore, the efficacy of PCSK9 inhibition in COVID-19 patients needs to be thoroughly evaluated in well-designed clinical trials. Furthermore, PCSK9 inhibitors are currently approved for managing dyslipidemia, and their use in COVID-19 would require careful consideration of potential drug interactions and safety concerns. Clinical trials are necessary to determine the optimal dosage, timing, and patient selection for PCSK9 inhibition in COVID-19.

#### Protection against ARDS

Emerging evidence suggests that PCSK9 may play a role in lung injury and inflammation, making it a potential target for mitigating ARDS and alleviating respiratory distress in COVID-19 patients. Recent studies have revealed that PCSK9 is not only involved in lipid metabolism but also has direct effects on lung cells and the respiratory system^53^. PCSK9 receptors have been found in the lung tissue, suggesting a possible role for PCSK9 in lung function and homoeostasis. Some preclinical studies have demonstrated that PCSK9 is implicated in lung inflammation and injury. The exact mechanisms by which PCSK9 may contribute to lung injury and inflammation are not fully understood. PCSK9 has been linked to the regulation of pro-inflammatory cytokines, such as interleukin-1 beta (IL-1β) and interleukin-18 (IL-18)^54^. These cytokines are known to play a significant role in the initiation and progression of lung inflammation. PCSK9 inhibition may reduce the production of these pro-inflammatory cytokines, thereby attenuating lung inflammation and injury. PCSK9 has been associated with increased oxidative stress, which can contribute to tissue damage and inflammation^55^. By inhibiting PCSK9, it might be possible to reduce oxidative stress and limit lung injury in COVID-19 patients. PCSK9 may influence immune cell function in the lungs. Immune cells play a crucial role in orchestrating the immune response during viral infections. PCSK9 inhibition might modulate the immune response in a way that promotes tissue repair and resolution of inflammation, potentially protecting against ARDS^56,57^. Considering the potential involvement of PCSK9 in lung injury and inflammation, PCSK9 inhibition may hold promise as a therapeutic strategy to protect against ARDS in severe COVID-19 cases. By reducing lung inflammation, PCSK9 inhibition could help preserve lung function and prevent the progression of respiratory failure. While the preliminary evidence is encouraging, it is essential to approach the potential use of PCSK9 inhibitors in COVID-19 with caution. ARDS in COVID-19 is a complex condition influenced by various factors, and its pathogenesis involves a combination of viral effects, immune dysregulation, and patient-specific factors.

#### Modulation of immune response

PCSK9 is not only involved in lipid metabolism but also in immune cell regulation. It has been shown to influence the differentiation and activation of certain immune cells, which are crucial for mounting an effective immune response against viral infections. By inhibiting PCSK9, it might be possible to fine-tune the immune response, leading to a better-balanced reaction against SARS-CoV-2 and potentially reducing the risk of immunopathology. Research has shown that PCSK9 inhibitors play a role in mitigating oxidative stress^58^. These inhibitors have been found to reduce the production of ROS in atherosclerotic plaques. This reduction in ROS production is significant because ROS can contribute to the oxidation of LDL cholesterol, endothelial dysfunction, and the activation of macrophages within the arterial walls^58^. These processes are proatherogenic, meaning they promote the development of atherosclerosis. Mitochondrial DNA damage is another hallmark of oxidative stress. Damage to mitochondrial DNA is often measured using specific markers like 8-OHdG, which is sensitive and specific to such damage. Studies have shown that PCSK9 inhibitors are effective in reducing the expression of 8-OHdG, indicating that they help protect mitochondrial DNA from oxidative damage^59^. This protection is essential because damage to mitochondrial DNA can lead to dysfunctional mitochondria, which, in turn, can further contribute to oxidative stress and inflammation within the arterial walls. Impaired autophagy has been associated with the worsening of vascular plaques, increased oxidative stress, and heightened inflammation^60^. When autophagy is compromised, cells struggle to efficiently eliminate unwanted substances and dysfunctional components, which can contribute to the development of atherosclerosis^60^. This is because atherosclerosis involves the accumulation of lipids, cellular debris, and inflammatory components within the arterial walls, which would typically be removed through effective autophagy. PCSK9 inhibitors have shown promise in enhancing autophagy in vascular tissue and macrophages, the immune cells within the arterial walls^58^. This enhancement of autophagy is significant in the context of atherosclerosis for several reasons. Autophagy plays a role in breaking down lipids, which are a fundamental component of the plaques that accumulate in atherosclerosis^58^. Enhanced autophagy can help in degrading these lipids, potentially reducing plaque formation. It also regulates inflammation. In atherosclerosis, inflammation contributes to plaque development. By promoting autophagy, PCSK9 inhibitors may help control inflammation within the arterial walls^58,59^. It is essential for maintaining cellular health and function. When autophagy is efficient, it can prevent cell damage and dysfunction, which are common in atherosclerotic conditions. Autophagy helps prevent the formation of macrophage foam cells, which are a key contributor to plaque development. By enhancing autophagy in macrophages, PCSK9 inhibitors may reduce the formation of these foam cells^58^. The protective effects of PCSK9 inhibitors in preventing atherosclerosis development are closely tied to their ability to enhance autophagy. By facilitating the removal of unwanted components, including lipids and inflammatory factors, they help maintain cellular health and reduce the conditions that promote plaque accumulation within arterial walls.

#### Potential antiviral effects

Recent studies have suggested that PCSK9 might be involved in the entry of certain viruses, including SARS-CoV-2, into host cells^61^. By inhibiting PCSK9, it may be possible to interfere with the virus’s entry process, limiting viral replication and spread. However, the extent of PCSK9’s direct involvement in viral entry and replication is still an area of ongoing research.

#### Beneficial effects on coagulation and thrombosis

Recent research has shed light on the potential role of PCSK9 in modifying platelet function, a critical component of blood clotting and the body’s response to vascular injury. Dyslipidemia, characterized by abnormal lipid levels in the bloodstream, can have a profound impact on the delicate balance of haemostasis and platelet reactivity^62^. Elevated levels of LDL-C have been linked to increased platelet reactivity and the heightened production of thromboxane, a molecule that promotes blood clot formation. Furthermore, high levels of circulating LDL-C are associated with increased oxidative stress, a condition characterized by the excessive production of ROS. This oxidative stress significantly contributes to inflammation-driven thrombosis, the pathological formation of blood clots within blood vessels^62^. Oxidized LDL (oxLDL), a product of oxidative stress due to high LDL-C concentrations, plays a central role in increasing platelet activation via CD36, a receptor involved in recognizing specific oxidized lipids and lipoproteins. CD36 is essential for processes like the clearance of apoptotic cells, responses to bacterial and fungal infections, and the uptake of LDL^62^. Activation of CD36 by oxLDL triggers inflammatory reactions that alter the progression of atherosclerosis, a condition characterized by the buildup of plaque in the arteries. Intriguingly, PCSK9 has been found to bind to CD36, which is a negative regulator of angiogenesis. This interaction between PCSK9 and CD36 results in the activation of platelets. However, the use of PCSK9 inhibitors has been shown to decrease platelet activity, mitigating their impact on thrombosis. Studies, such as the PCSK9—REACT study, have established a link between elevated PCSK9 levels and platelet activation, suggesting that PCSK9 can serve as a predictor of ischaemic events^63^. Research has demonstrated that PCSK9, through its interaction with the CD36 receptor, directly increases platelet activation and in vivo thrombosis^64^. Additionally, treatment with PCSK9 inhibitors has been shown to lead to reduced levels of LDL, oxLDL, and PCSK9 in patients with familial hypercholesterolaemia, which further supports the role of PCSK9 in platelet activation and thrombosis^65^. Furthermore, PCSK9’s interaction with CD36 activates enzymes and signalling pathways that are associated with the production of ROS, which can promote platelet activation and thrombosis. Some studies suggest that PCSK9 also plays a role in regulating CD36 and triglyceride metabolism^66^. Notably, PCSK9 inhibitors have been associated with a reduction in venous thromboembolism risk. This risk reduction is associated with baseline Lp(a) concentrations, suggesting that Lp(a) can serve as a marker for observation^67^. Moreover, PCSK9 inhibitors have been found to impact factors related to platelet activation and thrombosis, such as D-dimer, fibrinogen, and plasminogen activator inhibitor-1 (PAI-1)^68^. Additionally, they have been shown to raise HDL cholesterol levels, which can indirectly prevent platelet aggregation by affecting platelet membrane cholesterol. PCSK9 is also associated with cellular apoptosis in vascular smooth muscle and endothelial cells. The death of these cells can promote thrombosis through the production of procoagulant microparticles^69^. Interestingly, platelets have been found to secrete PCSK9 after activation in the presence of LDL, which can enhance platelet aggregation and thrombus formation.

### Safety considerations

Safety considerations and possible risks are critical factors that need to be carefully evaluated when considering the use of PCSK9 inhibitors in the context of COVID-19 or any other medical condition. While PCSK9 inhibitors have shown promising results in lipid management and potential benefits in other areas, it is essential to understand and address potential safety concerns before implementing them in clinical practice. PCSK9 inhibitors are highly effective in reducing LDL cholesterol levels, which is their primary intended effect. However, excessively low LDL cholesterol levels could have unintended consequences. Cholesterol is essential for various cellular functions, including hormone synthesis and cell membrane integrity. Therefore, careful monitoring of cholesterol levels and appropriate dose adjustments are crucial to maintaining a balance between LDL cholesterol reduction and overall health.

PCSK9 inhibitors can interact with other medications, including immunosuppressants and anticoagulants, potentially affecting their effectiveness or safety. Drug interactions should be thoroughly evaluated, especially in COVID-19 patients receiving multiple medications for their condition. Healthcare providers should be cautious about potential interactions and adjust the treatment plan accordingly.

Safety in Specific Populations: Special attention should be given to the safety of PCSK9 inhibitors in specific populations, such as pregnant or breastfeeding individuals, children, and patients with severe liver or kidney impairment. The safety profile of PCSK9 inhibitors may vary in these groups, and their use should be approached cautiously, considering individual risks and benefits. PCSK9 inhibitors are biological drugs that can induce an immune response in some patients. This immunogenicity could potentially reduce the drug’s effectiveness over time or lead to allergic reactions. Monitoring for any signs of immune reactions or decreased efficacy is important to ensure optimal patient outcomes. While PCSK9 inhibitors have demonstrated favourable safety profiles in clinical trials, their long-term safety in the context of COVID-19 or other chronic conditions needs further investigation. Continuous monitoring and post-marketing surveillance are essential to assess any potential rare or long-term adverse events. PCSK9 inhibitors are biological drugs and can be expensive, which may limit access for some patients. The cost-effectiveness of these medications should be carefully evaluated, considering potential benefits and risks. PCSK9 inhibitors may influence the immune response, and this could have implications for COVID-19 patients. It is essential to understand how PCSK9 inhibition may affect the body’s ability to respond to viral infections, especially in critically ill patients. While PCSK9 inhibitors are designed to target PCSK9 specifically, there is always a possibility of off-target effects on other proteins or biological pathways. Understanding these potential off-target effects is critical for assessing the overall safety profile of PCSK9 inhibitors. Although PCSK9 is primarily known for its role in regulating lipid metabolism in the liver, recent research has revealed its presence in non-hepatic tissues, including the brain. There is emerging evidence that links PCSK9, both in terms of protein levels and genetic variations within the PCSK9 gene, to mood disorders and related traits, such as depressive symptoms and neuroticism^70^. Mendelian randomization studies, which utilize genetic risk scores based on PCSK9 gene variants, have suggested an association between PCSK9 and an increased risk of major depressive disorder, although not with neuroticism^71^. These findings imply that PCSK9 may play a role in the development of major depressive disorder. In vivo experiments have also provided insights into the potential impact of PCSK9 on the brain. Overexpression of LDLR, a protein targeted for degradation by PCSK9, in the brains of mice triggered neuroinflammatory responses, suggesting that inhibiting PCSK9 might lead to neuroinflammation^72^.

Using PCSK9 inhibition as a therapeutic approach for COVID-19 presents several challenges and potential limitations. As of my last knowledge update in September 2023, clinical data supporting its use were limited^58^. This lack of data means that the clinical impact and safety of PCSK9 inhibitors for COVID-19 remain uncertain. COVID-19 is a multifaceted disease with various underlying mechanisms. PCSK9 inhibition might address some aspects of the disease, but it is unlikely to serve as a comprehensive solution for all cases. The risk-benefit profile of PCSK9 inhibitors for COVID-19 needs careful evaluation, considering that COVID-19 primarily affects the respiratory system and may involve different pathways compared to their typical use for lipid control. Like any medication, PCSK9 inhibitors can have side effects, some of which may not be apparent when used for cholesterol management. These potential side effects must be taken into account. Cost and accessibility are crucial factors. PCSK9 inhibitors can be expensive, and ensuring broad access during a global pandemic could be challenging. The development of PCSK9 inhibitors specifically for COVID-19 would require rigorous testing and regulatory approvals, making the process time-consuming and costly. COVID-19 strains vary, and the effectiveness of a treatment may differ between strains. Ensuring that PCSK9 inhibition works across various strains is vital for its success in treating COVID-19.

### Future directions

Rigorous clinical trials are needed to assess the safety and efficacy of PCSK9 inhibitors in COVID-19 patients, especially those at high risk for severe outcomes. Large-scale randomized controlled trials can help determine the impact of PCSK9 inhibition on reducing inflammation, mitigating cardiovascular complications, and improving overall survival in COVID-19 patients. Research can focus on determining the optimal timing and dosage of PCSK9 inhibitors during COVID-19 infection to achieve maximum benefits while minimizing potential risks. This may involve studying different treatment regimens and their impact on lipid metabolism, inflammation, and thrombosis in the context of the disease. Investigating the potential benefits of combining PCSK9 inhibitors with other therapeutics used in COVID-19 management, such as antiviral drugs and anticoagulants, can offer synergistic effects and improved patient outcomes. Studies exploring the safety and efficacy of combination therapies could provide valuable insights. Understanding the impact of PCSK9 inhibition on the immune response in COVID-19 patients is crucial. Further research can explore the effects of PCSK9 inhibitors on immune cell function, viral clearance, and vaccine responses to ensure that these medications do not compromise the body’s ability to fight the infection. Long-term safety data are essential to assess the potential risks associated with prolonged PCSK9 inhibitor use. Observational studies and real-world evidence can provide insights into any rare or delayed adverse events and inform clinical practice guidelines. Identifying specific patient subgroups that may benefit the most from PCSK9 inhibition in COVID-19 or other conditions can enhance treatment precision. Personalized medicine approaches can optimize treatment strategies and improve outcomes based on individual patient characteristics and risk profiles. PCSK9’s role in inflammation, immune modulation, and thrombosis is not yet fully understood. Further research is needed to elucidate the molecular mechanisms underlying these non-lipid-related functions, paving the way for targeted therapeutic interventions. Beyond COVID-19, PCSK9 inhibitors have the potential to benefit patients with other inflammatory and cardiovascular conditions. Research can explore the safety and efficacy of PCSK9 inhibitors in diseases such as atherosclerosis, rheumatoid arthritis, and other immune-mediated disorders. Developing new PCSK9 inhibitors with improved pharmacokinetic profiles, alternative routes of administration, and enhanced efficacy may offer additional treatment options and expand the utility of PCSK9 inhibition in various clinical scenarios. Conducting health economic analyses can assess the cost-effectiveness of PCSK9 inhibitors in different patient populations, helping policymakers make informed decisions about resource allocation and reimbursement.

## Conclusion

In conclusion, PCSK9 inhibition represents a compelling and multifaceted area of research with potential implications in the management of COVID-19 and other medical conditions. The interplay between PCSK9 and various physiological processes, including lipid metabolism, inflammation, and thrombosis, offers a promising avenue for therapeutic intervention. In the context of COVID-19, PCSK9 inhibitors hold promise for their potential to attenuate the cytokine storm, protect against ARDS, and mitigate cardiovascular complications. By reducing LDL cholesterol levels and modulating immune and inflammatory responses, PCSK9 inhibition could play a role in improving patient outcomes and potentially reducing the severity of the disease. However, it is essential to approach the use of PCSK9 inhibitors in COVID-19 and other conditions with caution, considering potential safety considerations and drug interactions. Rigorous clinical trials are necessary to validate the efficacy and safety of PCSK9 inhibitors in specific patient populations and to determine the optimal timing and dosing of these medications.

## Ethical approval

Ethics approval was not required for this Review Article.

## Consent

Informed consent was not required for this Review Article.

## Sources of funding

The authors received no specific funding for this manuscript.

## Author contribution

Concept: J.M., H.A., N.I., V.K., U.N.K. Data collection: C.P.K., F.P., H.A, Design: S.K., M.T.H., D.K., D.R., F.P. Data analysis: S.K., A.M. Writing: C.P.K., F.P., H.A., S.K., M.T.H., D.K., D.R., F.P., S.K., J.M. Supervision: A.M., J.M.

## Conflicts of interest disclosure

The authors have no conflict of interests.

## Research registration unique identifying number (UIN)

Not applicable.

## Guarantor

Amin mehmoodi.

## Data availability statement

Not applicable.

## Provenance and peer review

Not invited.
